# Splenomegaly, Cardiomegaly, and Osteoporosis in a Child with Gaucher Disease

**DOI:** 10.1155/2011/564868

**Published:** 2011-09-29

**Authors:** J. J. Sheth, C. M. Ankleshwaria, M. A. Mistri, N. Nanavaty, S. J. Mehta

**Affiliations:** ^1^Institute of Human Genetics, FRIGE House, Jodhpur Gam Road, Satellite, Ahmedabad 380015, India; ^2^Opp. Sanghvi High School, Naranpura, Ahmedabad 380014, India; ^3^Usha Deep Children Neurology and Epilepsy Clinic, 3rd Floor, Mansarovar Complex, Naranpura, Ahmedabad 380014, India

## Abstract

A 15-month-old girl, born to the consanguineous parents, was referred with the sign of massive splenomegaly associated with thrombocytopenia and anemia. Plasma Chitotriosidase estimation was carried out as a screening test and was found to be normal with reduced activity of **β**-glucosidase in leucocytes suggestive of Gaucher disease. At the age of 4 years, severe osteoporosis and cardiomegaly with pulmonary congestion were observed in the child. Molecular analysis for GBA gene has revealed homozygous status for L444P (c.1448C) in the proband, whereas parents and two elder sisters were found to be heterozygote. Prenatal study during the fourth pregnancy was carried out from cultured chorionic villi for **β**-glucosidase, which was in the carrier range. Further confirmation of the carrier status was carried out from amniotic fluid DNA and was found to be heterozygous for L444P (c.1448C) in the *GBA* gene. This case demonstrates that children with the sign of splenomegaly with anemia and thrombocytopenia need to be screened for Gaucher disease, and molecular study can further help to confirm the heterozygous status, where prenatal study by enzyme investigation demonstrate heterozygous condition.

## 1. Introduction

Gaucher disease (GD) is an autosomal recessive sphingolipid disorder resulting from the accumulation of glucocerebroside in the cells of macrophage-monocyte system as a result of a deficiency in lysosomal enzyme *β*-glucosidase [glucocerebrosidase, E.C. 3.2.1.45], which is encoded by the GBA gene on chromosome-1 [1]. The three main clinical types have been delineated according to the absence (type 1, nonneuronopathic) or the presence (type 2, acute neuronopathic, and type 3, sub acute neuronopathic) of neurological involvement [2]. Glucosylceramide, the accumulated glycolipid, is primarily derived from the phagocytosis and degradation of senescent leukocytes and, to a lesser extent, from erythrocyte membranes. The glycolipid storage gives rise to the characteristic Gaucher cells, which are typically present in the bone marrow, liver, spleen, lungs, and other organs. This contributes to pancytopenia, massive hepatosplenomegaly, and at times diffuse infiltrative pulmonary disease. However, signs like anemia, severe splenomegaly, and hepatomegaly were observed more frequently in younger patients [3].

## 2. Case Report

**Figure 1:**
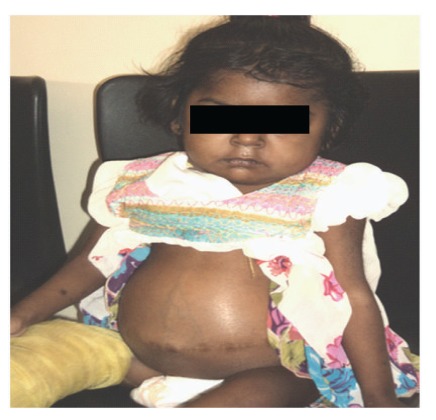
Proband with hepatomegaly at 4 years of age.

**Figure 2:**
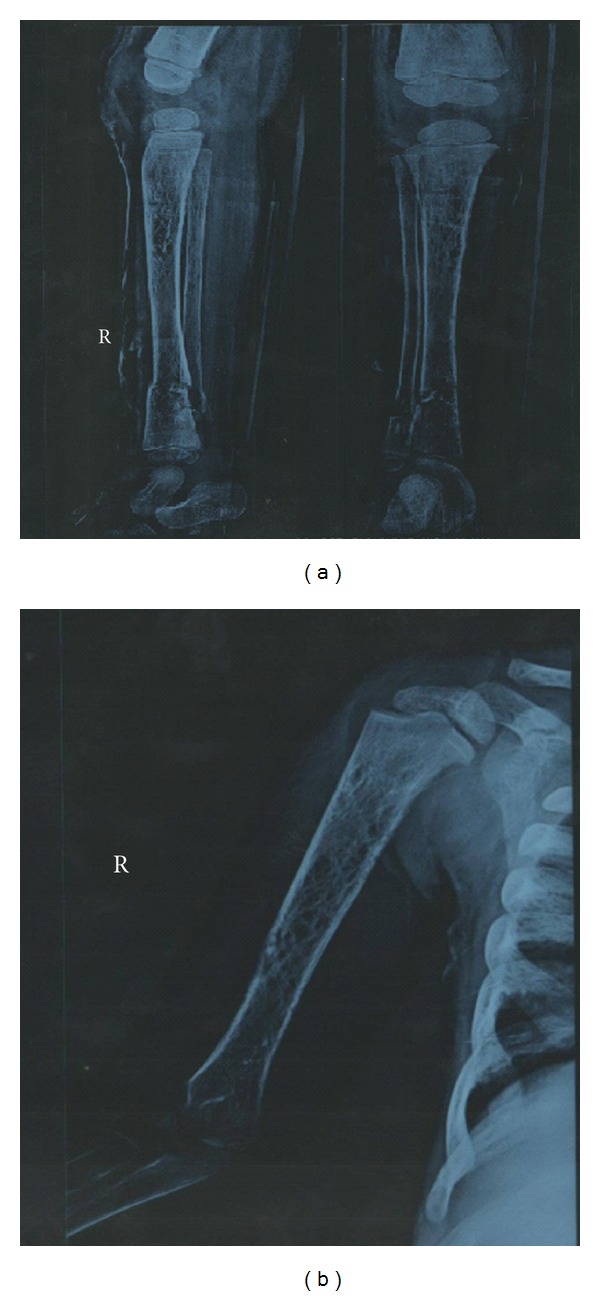
(a) X-ray of RT T/F AP/LAT shows osteoporosis and fractures. (b) X-ray of RT humerus shows severe osteoporosis.

A 15-month-old female child was referred to our centre with the clinical sign of massive splenomegaly associated with thrombocytopenia and anemia. She was delivered after full-term normal pregnancy. She had seizure immediately after 2 hours of birth and was kept under observation for a day. Development of the child was normal till 1 year of age. Later on, her parents noticed sporadic crying and regression of milestones especially when she stopped walking. Her two elder sisters were healthy, and there was no family history of blood transfusion, splenectomy, and recurrent jaundice. She was referred for plasma chitotriosidase estimation at the age of 15 months, which was 64.12 nmol/hr/mL plasma (normal range: 28.66–62.94 nmol/hr/mL plasma). Ultrasound scanning at 15 months showed the spleen of about 11-12 cm. No active bleeding, hepatomegaly, or lymphadenopathy was observed. X-ray of the pelvis with lower limbs was found to be normal. Subsequently, colour Doppler was carried out, which had shown huge splenomegaly with normal splenic echotexture and splenic vein measuring 4 mm at splenic hilum. There was no evidence of periportal varices observed, and fundoscopy examination was found normal. On systemic examination, central nervous system (CNS) report showed consciousness with normal eye movements. She had no focal deficit, power was normal, mild hypotonia with deep tendon reflex (DTR)-brisk was noted. Proband was referred once again at the age of 2.5 years due to difficulty in walking with persistence of a large abdomen and her histopathology examination from liver biopsy also showed characteristic Gaucher cells occupying the sinusoids, and the spleen biopsy section revealed large cells with fibrillary cytoplasm and small eccentric nuclei populating red pulp. Plasma chitotriosidase study was reconfirmed and was found normal 32.00 nmol/hr/mL plasma, and *β*-glucosidase activity from leucocytes was low 3.8 nmol/hr/mg protein (normal range: 8.0–32.0 nmol/hr/mg protein) suggesting GD in the proband. At the age of 4 years, due to septicemia and bleeding from the mouth, proband was operated for splenectomy. At this time, X-ray of the chest posterior-anterior view showed cardiomegaly with pulmonary congestion and electrocardiogram (ECG) report revealed mild left ventricular systolic dysfunction with 40–50% of left ventricular ejaculation function. Ultrasound scanning at this time showed an enlarged liver measuring 15.5 cm in size (Figure 1). X-ray of the right tibia/fibula anteroposterior and lateral view revealed osteoporosis and fracture (Figure 2(a)) and X-ray of the right humerus revealed severe osteoporosis with cortical irregularity and fracture seen along midshaft of the humerus (Figure 2(b)). Hematogram investigation at this time showed Hb-6.7 (N.R.: 13.5–18.0 g/dL), total WBC count-29,484 (N.R.: 4,000–11,000/cmm), and platelet count-45,000 (N.R.: 150,000–450,000/cmm).

Further confirmation of the disease was carried out by molecular analysis of *GBA* gene. This has shown homozygous status for L444P (c.1448C), confirming GD. Two unaffected elder sisters and parents revealed heterozygous status for L444P (c.1448C) mutation. During the fourth pregnancy cultured chorionic villus study was carried out for *β*-glucosidase, which was in the carrier range 139.58 nmol/hr/mg protein with control value of 328.3 nmol/hr/mg protein. Further confirmation was carried out from amniotic fluid DNA, which has confirmed heterozygous status of the fetus for the said mutation.

## 3. Discussion

The most common signs and symptoms noted in GD are splenomegaly (95%), hepatomegaly (87%), radiological bone disease (81%), thrombocytopenia (50%), anemia (40%), growth retardation (34%), bone pain (27%), and bone crisis (9%). A skeletal manifestation is found more often in older children [3]. The femoral head and the femoral shaft are by far the most frequently involved sites in bone crisis. However, these episodes occur with some frequency in humeral heads, vertebral bodies, and ischium of pelvis [4]. In the present case, all these symptoms were observed and in addition osteoporosis and fracture in lower shaft tibia/fibula, severe osteoporosis with cortical irregularity and fracture were seen along the midshaft of the humerus. Cardiomegaly with pulmonary congestion was also observed at this time, and it is likely to occur due to frank infiltration of lungs by Gaucher cells [4].

Plasma chitotriosidase, which acts as a screening marker and shows marked elevated level in Gaucher disease, was found to be normal in present case [5]. This can be explained by the fact that 5-6% of the population who lack this enzyme as a result of genetic deficiency due to an expressional mutation in the human chitotriosidase gene that occurs with high polymorphic frequency and approximately one-third of patients with GD are heterozygous for this null allele, and thus the extent to which GD may increase the activity of chitotriosidase in the plasma is reduced in these individuals [6, 7]. Although *β*-Glucosidase activity in leucocytes or fibroblasts is the confirmative test for GD followed by mutation identification in GBA gene [8]. Our study is in accordance with this observation with significantly low activity of the enzyme in leucocytes followed by identification of L444P mutation in the proband. Identification of the genotype may help in predicting phenotypic expression, therapeutic response, and carrier screening for genetic counseling. Presence of L444P (c.1448C) homozygous status in the proband was useful in detecting carrier status in both parents, two unaffected sisters, and during prenatal diagnosis. This is one of the most common mutation observed in non-Jewish populations with a frequency of 31.43% and resulted in substitution of proline for leucine at position 444(L444P) [9]. This mutation was first found in neuronopathic form (Type II and type III) but later on it was also found to be associated with the nonneuronopathic form (Type I) [10]. 

It has been reported that nonneuronopathic GD is the most prevalent form (94%) and is differentiated from the acute neuronopathic (1%) and chronic neuronopathic (5%) forms by the absence of central nervous system involvement [3]. In the present report, hepatosplenomegaly, anemia, thrombocytopenia, osteoporosis, cardiomegaly, mild hypotonia, and homozygous for L444P (c.1448C) mutation and normal central nervous system examination suggest Type-I Gaucher disease.

In such cases the long-term clinical picture is critical in light of data demonstrating that the efficacy of enzyme replacement therapy (ERT) with placental-derived preparation, alglucerase or the recombinant form, imiglucerase and substrate reduction therapy (e.g., miglustat) can prevent progressive manifestations of GD and completely or partly ameliorates disease-associated anemia, thrombocytopenia, organomegaly, bone pain, and bone crises [11]. 

As per our knowledge, this is the only case study from India where screening to confirmative molecular analysis has been carried out in the family. It clearly demonstrates that primary screening by plasma chitotriosidase followed by confirmative enzyme study of *β*-Glucosidase together with mutation identification can help the family to corroborate the prenatal and postnatal diagnosis for an early therapeutic approach, before irreversible clinical manifestation occurs.
